# Cancer patients with COVID-19: does prior nutritional risk associated with cancer indicate a poor prognosis for COVID-19?

**DOI:** 10.31744/einstein_journal/2023AO0172

**Published:** 2023-03-07

**Authors:** Livia Costa de Oliveira, Karla Santos da Costa Rosa, Aline Pereira Pedrosa, Naira Freire da Silva, Lara Azevedo dos Santos, Emanuelly Varea Maria

**Affiliations:** 1 Instituto Nacional de Câncer Rio de Janeiro RJ Brazil Instituto Nacional de Câncer , Rio de Janeiro , RJ , Brazil .

**Keywords:** COVID-19, Coronavirus, Coronavirus infections, Malnutrition, Nutritional status, Neoplasms, Prognosis

## Abstract

**Objective:**

To verify whether the presence of related nutritional risk indicators prior to COVID-19 diagnosis is associated with poor survival in patients with cancer.

**Methods:**

We retrospectively analyzed the data of hospitalized cancer patients who tested positive for COVID-19 between March 2020 and February 2021. Nutritional risk was defined as the presence of one of the following characteristics: body mass index <20kg/m ^2^ , scored Patient-generated Subjective Global Assessment ≥9 points or classification B, albumin level <3.5g/dL, and C-reactive protein level ≥10mg/L, evaluated between 7 and 60 days prior to the date of patient inclusion. The endpoint measure was all-cause mortality within 30 days of COVID-19 diagnosis.

**Results:**

A total of 253 patients were included, most of whom were elderly (62.4%) and female (63.6%). Overall, 45.4% of the patients were at nutritional risk. Survival was significantly lower in patients at nutritional risk (8 days; interquartile range [IQR]: 3-29) than in patients not at nutritional risk (16 days; IQR: 6-30) (p<0.001). The presence of prior nutritional risk was associated with increased 30-day mortality (HR: 1.42; 95%CI: 1.03-1.94), regardless of age, gender, tumor site or stage, and other risk factors, and the model had good discrimination accuracy (concordance statistic: 0.744).

**Conclusion:**

The presence of prior nutritional risk indicators is related to poor prognosis in patients with cancer and COVID-19, emphasizing the importance of nutritional care, notably during this pandemic.

**Figure:**
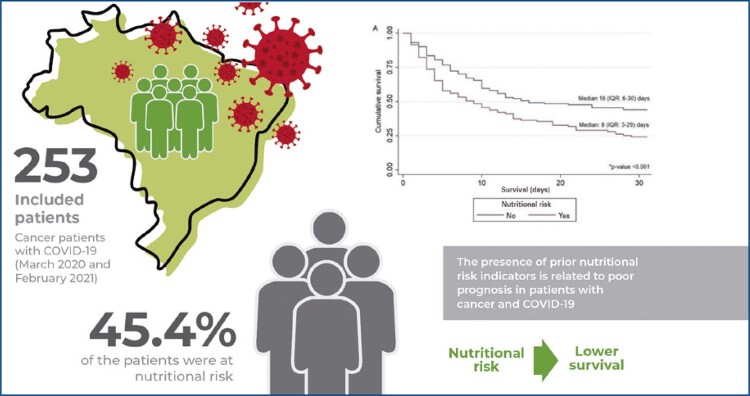

## INTRODUCTION

Severe acute respiratory syndrome coronavirus 2 (SARS-CoV-2), resulting in coronavirus disease 2019 (COVID-19), emerged as a global pandemic owing to its rapid transmission and the susceptibility of the population, posing unprecedented challenges to patients and healthcare systems. ^( 1 )^ More severe complications and deaths have been reported among older patients and individuals with underlying conditions, such as cardiovascular, liver, and kidney disease, and cancer. ^( 2 , 3 )^

Patients with cancer are generally assumed to be at high nutritional risk ^( 4 )^ and appear to be at increased risk of adverse outcomes from COVID-19 infection. ^( 5 - 8 )^ In addition, patients with COVID-19 are at high risk of being malnourished, with poor nutritional status having been associated with progression to severe disease and adverse effects ( *e.g.,* intensive care unit admission, mechanical ventilation requirement, and mortality). ^( 9 - 12 )^

The prevalence of nutritional risk in hospitalized patients with COVID-19 have been described by different screening methods. ^( 13 - 16 )^ Despite the possible contribution of poor nutritional status to the acquisition and unfavorable outcomes of COVID-19 infection, there are several knowledge gaps in clinical nutrition applicable to the COVID-19 pandemic, and there is very limited data on the prognostic and predictive role of nutritional risk in these patients. ^( 17 - 19 )^ The interactions of COVID-19 with pre-existing malignancy and nutritional status are poorly described. Indeed, to the best of our knowledge, only one study has evaluated the prognostic role of nutritional risk in a subsample of hospitalized adult patients with cancer and COVID-19. ^( 20 )^

Given that nutritional impairment is common in adult patients with cancer, ^( 21 - 24 )^ it follows that the deleterious consequences of malnutrition could impact the patients’ COVID-19 prognosis. ^( 12 , 17 )^ Additionally, given the global prevalence of cancer and the high transmissibility of SARS-CoV-2, an understanding of the disease course of COVID-19 and factors influencing clinical outcomes in patients with cancer is urgently needed. ^( 6 )^

In the context, we hypothesize that, among patients with cancer who are diagnosed with COVID-19, mortality incidence differs between patients who are at nutritional risk and those who are not at nutritional risk.

## OBJECTIVE

To verify whether the presence of related nutritional risk indicators prior to COVID-19 diagnosis is associated with poor survival in patients with cancer.

## METHODS

### Patients and study design

A hospital-based retrospective observational cohort study was conducted with data extracted from the electronic medical records of all the patients hospitalized for COVID-19 at the reference cancer institute between March 2020 and February 2021.

**Figure 1:**
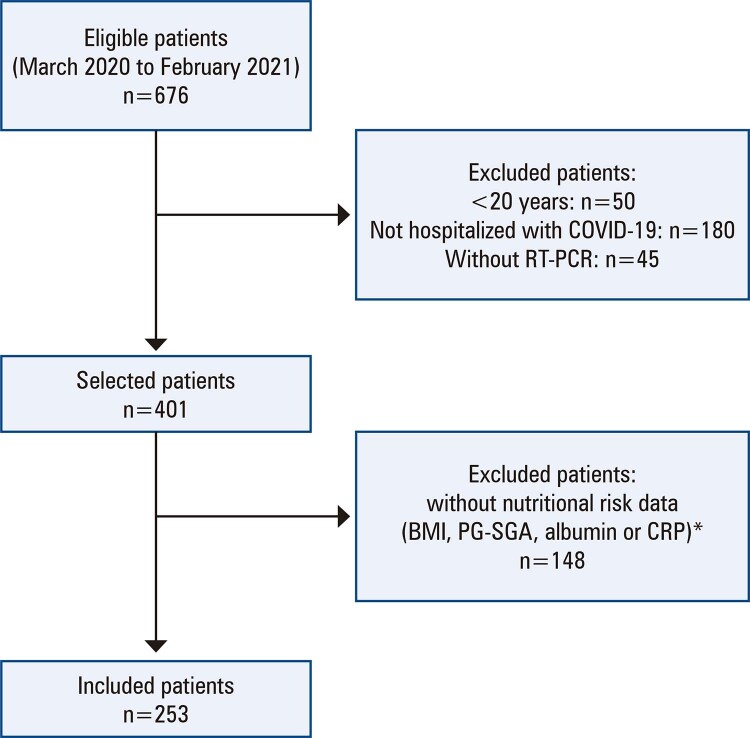
Flowchart of the study sample

Inclusion criteria were: age ≥20 years; confirmed diagnosis of malignant neoplasm (active treatment or exclusive palliative care), regardless of tumor site and time of diagnosis; hospitalized for COVID-19, confirmed by a positive reverse-transcriptase polymerase chain reaction (RT-PCR) test for SARS-CoV-2. ^( 1 )^ Patients without a positive RT-PCR test (n=45), who were not admitted to hospital during infection (n=180), and with missing nutritional risk data (n=148) were excluded ( Figure 1 ). None of the patients had received any dose of vaccine against COVID-19, as there was none available at the time of participant recruitment.

* There was no statistical difference in the sample studied when compared with the missing data in relation to age (p=0.132), gender (p=0.354), primary tumor site (p=0.247), and Karnofsky Performance Status (KPS) or Eastern Cooperative Oncology Group performance status (ECOG-PS) (p=0.157).

RT-PCR: reverse transcription polymerase chain reaction; BMI: body mass index; PG-SGA: Patient-generated Subjective Global Assessment; CRP: C-reactive protein.

The study was approved by the Research Ethics Committee of the *Instituto Nacional de Câncer* (INCA), CAAE: 31053220.0.0000.5274; # 4.511.910. Informed consent was waived because data were extracted from medical records retrospectively (non-invasive observational study).

Demographic and clinical data were collected at hospital admission whenever a patient had a positive SARS-CoV-2 test result.

Patients were followed up from the date of hospital admission until 30 days after inclusion in the study. Outcomes were monitored up to March 31, 2021, the final date of follow-up.

### Data collection

#### Nutritional risk

The main independent variable evaluated was the presence of nutritional risk (yes/no). Nutritional risk was defined as the presence of one of the following characteristics: body mass index (BMI) <20kg/m ^2^ , score Patient-Generated Subjective Global Assessment (PG-SGA) ≥9 points or global classification B (suspected malnutrition), ^( 25 )^ albumin level <3.5g/dL, and C-reactive protein (CRP) level ≥10mg/L. ^( 4 , 26 )^

Assuming a mean delay of approximately one week between COVID-19 infection and a positive diagnosis, the nutritional risk data analyzed were from 7 to 60 days prior to the date of the patients’ inclusion (median: 16; interquartile range [IQR]: 9-30 days) and were available for 253 of the 401 patients selected for the study. There was no statistical difference between the patients included in the sample and the ones for whom nutritional risk data were missing: age (p=0.132), gender (p=0.354), primary tumor site (p=0.247) and Karnofsky Performance Status or ECOG-Performance Status (p=0.157).

#### Other covariates

The demographic data collected from the enrolled patients were: age (<60 or >60 years) and gender (male or female). The clinical characteristics included cancer diagnosis (site of primary cancer: gynecological, gastrointestinal tract, breast, head and neck, lung, bone and connective tissue, or other); tumor stage at the time of inclusion in the study (stages I and II [no metastasis] or stages III and IV [local or distant metastasis]); number of metastasis sites (<1 or >2); lung metastasis at the time of inclusion in the study (in patients without primary lung cancer) (yes or no); surgery and/or chemotherapy and/or radiation therapy within 60 days of admission (yes or no); and preexisting comorbidities ( *diabetes mellitus* , systemic arterial hypertension, cardiovascular disease or chronic obstructive pulmonary disease [yes or no]). Performance status data were obtained by administering the simple 6-item ECOG-PS scale that ranges from 0 (normal activity) to 5 (dead), ^( 27 , 28 )^ or the 11-point KPS scale, with scores ranging from 100 (normal activity) to 0 (dead). ^( 29 )^ These scales were converted and categorized as ECOG-PS score ≥3 or KPS score ≤40% (yes or no), as suggested by Ma et al. ^( 30 )^

#### Outcome

The endpoint measure was all-cause mortality within 30 days of COVID-19 diagnosis. Survival was assessed longitudinally, counting from the date of the positive COVID-19 test until death. For the analyses, the survival times were censored on the patients who were alive after this endpoint.

#### Statistical analysis

Statistical analyses were performed using Stata 13.1 (Stata Corp., College Station, Texas, USA). Statistical significance was set at p<0.05.

The Kolmogorov-Smirnov test was used to assess the distribution of variables. Numerical variables were described as medians with iterquartile range (IQR) IQR (25 ^th^ and 75 ^th^ percentiles) and categorical variables were described as absolute (n) and relative frequencies (%). Proportions were compared using the χ ^2^ test, and medians were compared using the corresponding non-parametric test, the Mann-Whitney U test.

The Kaplan-Meier method and the log-rank test were used to compare survival according to groups. The Cox proportional hazard model was used to assess the predictive ability of nutritional risk, using hazard ratios (HRs) with 95% confidence interval (95%CI). All the factors with p<0.20 in the univariate analysis were included in the multivariate analysis. The final model was produced using the backward selection, and the variables with p value <0.05 were maintained.

Harrell’s C-statistic ^( 31 )^ with 95%CI was applied to assess the discriminatory power of nutritional risk in predicting 30-day mortality; 0.50 indicates the outcome as well as chance, 0.70 to <0.80: good discrimination, 0.80 to <0.90: excellent discrimination, 0.90 to <1.00: outstanding discrimination, and 1.00: perfect prediction. ^( 32 )^

## RESULTS

A total of 253 patients with cancer and COVID-19 were included in the analysis. The patients were predominantly older (≥60 years, 62.4%) and female (63.6%). Breast was the most prevalent tumor site (19.8%), followed by gastrointestinal tract (18.6%), and 82.6% of the patients were at stage III or IV ( Table 1 ).

**Table 1:** Patients’ characteristics with cancer and COVID-19 diagnoses according to nutritional risk

Variables	Total	Nutritional risk		

		Yes (n=115; 45.4%)	No (n=138; 54.6%)	p value
Age (years)*				
<60	95 (37.6)	46 (48.4)	49 (51.6)	0.462
> 60	158 (62.4)	69 (43.7)	89 (56.3)	
Median (IQR) ^†^	63 (54-70)	61 (53-70)	63 (55-70)	0.443
Gender*				
Male	92 (36.4)	36 (39.1)	56 (60.9)	0.127
Female	161 (63.6)	79 (49.1)	82 (50.9)	
Primary tumor site*				
Breast	50 (19.8)	25 (50.0)	25 (50.0)	0.564
Gastrointestinal tract	47 (18.6)	27 (57.4)	20 (42.6)	0.044
Gynecological	36 (14.2)	21 (58.3)	15 (41.7)	0.021
Lung	22 (8.7)	10 (45.4)	12 (54.6)	0.189
Head and neck	14 (5.5)	6 (42.9)	8 (57.1)	0.074
Bone and connective tissue	9 (3.6)	6 (66.7)	3 (33.3)	<0.001
Others ^‡^	75 (29.6)	20 (26.7)	55 (73.3)	<0.001
Cancer stage*				
I or II	44 (17.4)	14 (31.8)	30 (68.2)	0.046
III or IV	209 (82.6)	101 (48.3)	108 (51.7)	
Number of metastasis*				
< 1	170 (67.2)	69 (40.6)	101 (59.4)	0.056
> 2	83 (32.8)	46 (55.4)	37 (44.6)	
Lung metastasis*				
No	205 (81.0)	90 (43.9)	115 (56.1)	0.306
Yes	48 (19.0)	25 (52.1)	23 (47.9)	
Surgery within 60 days*				
No	226 (89.3)	106 (46.9)	120 (53.1)	0.181
Yes	27 (10.7)	9 (33.3)	18 (66.7)	
Chemotherapy within 60 days*				
No	158 (62.4)	79 (50.0)	79 (50.0)	0.061
Yes	95 (37.6)	36 (37.9)	59 (62.1)	
Radiotherapy within 60 days*				
No	229 (90.5)	102 (44.5)	127 (55.5)	0.368
Yes	24 (9.5)	13 (54.2)	11 (45.8)	
Comorbidities				
*Diabetes mellitus* *				
No	199 (79.0)	93 (46.7)	106 (53.3)	0.497
Yes	53 (21.0)	22 (41.5)	31 (58.5)	
Hypertension ^*^				
No	138 (54.8)	62 (44.9)	76 (55.1)	0.804
Yes	114 (45.2)	53 (46.5)	61 (53.5)	
Cardiovascular disease*				
No	231 (91.7)	103 (44.6)	128 (55.4)	0.269
Yes	21 (8.3)	12 (57.1)	9 (42.9)	
COPD*				
No	241 (95.6)	112 (46.5)	129 (53.5)	0.211
Yes	11 (4.4)	3 (27.3)	8 (71.7)	
ECOG-PS 3-4 or KPS ≤40%*				
No	110 (43.8)	41 (37.3)	69 (62.7)	0.022
Yes	141 (56.2)	73 (51.8)	68 (48.2)	
Death within 30 days*				
No	89 (35.2)	28 (31.5)	61 (68.5)	0.001
Yes	164 (64.8)	87 (53.0)	77 (47.0)	
Hospital discharge*				
No	166 (65.6)	83 (50.0)	83 (50.0)	0.034
Yes	87 (34.4)	32 (36.8)	55 (63.2)	

* χ ^2^ test; ^†^ Mann-Whitney U test; ^‡^ Central nervous system, kidney and urinary tract, male genital organs, peritoneum, mediastinum, unrecognized site, and head and neck.

IQR: interquartile range; COPD: chronic obstructive pulmonary disease; ECOG-PS: Eastern Cooperative Oncology Group performance status; KPS: Karnofsky Performance Status.

**Figure 2:**
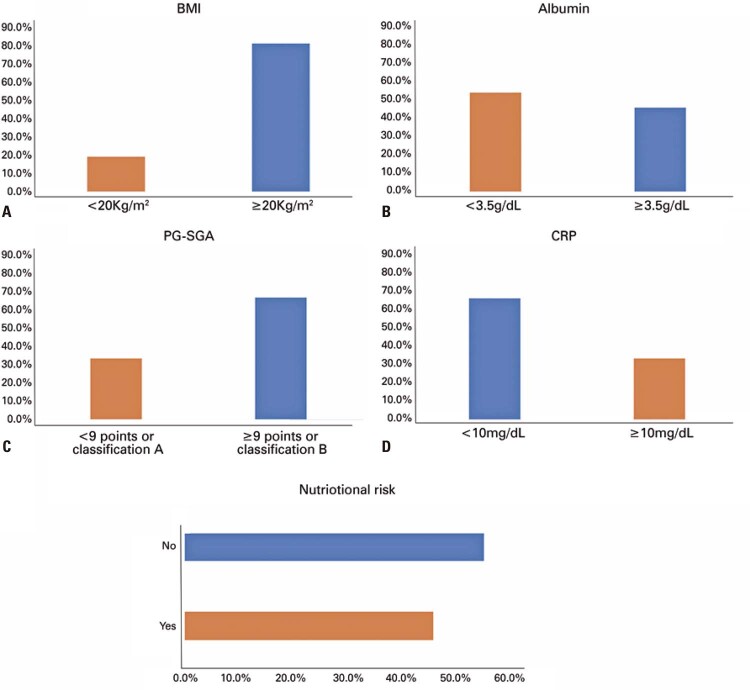
The prevalence of nutritional risk factors parameters among patients with cancer and COVID-19 diagnoses

BMI: body mass index; PG-SGA: Patient-generated Subjective Global Assessment; CRP: C-reactive protein.

The proportion of patients with PG-SGA score ≥9 points or classification B was 66.7%. In addition, 54.1% had hypoalbuminemia, 19.0% had BMI <20kg/m ^2^ , and 33.5% had CRP >10mg/L. Overall, 45.4% of the patients presented some characteristics related to nutritional risk ( Figure 2 ).

The prevalence of nutritional risk varied according to the assessment criteria and the clinical and demographic characteristics. The median time from confirmed COVID-19 diagnosis to the study endpoints (death or discharge from hospital) was 11 days (IQR: 4-30). At analysis (March 31, 2021), 166 (65.6%) of the patients had died, all within 30 days of COVID-19 diagnosis. There was a higher proportion of deaths among the patients at nutritional risk than among the patients not at nutritional risk (p=0.001) ( Table 1 ). It is worth noting that, in our study, it was enough for patients to fulfill just one of the criteria used to identify nutritional risk, and that even though 85 patients fulfilled one of these criteria, 15 fulfilled two, 10 fulfilled three, and 5 fulfilled all four, there was no statistical difference in the proportion of deaths across these groups (data not shown).

**Figure 3:**
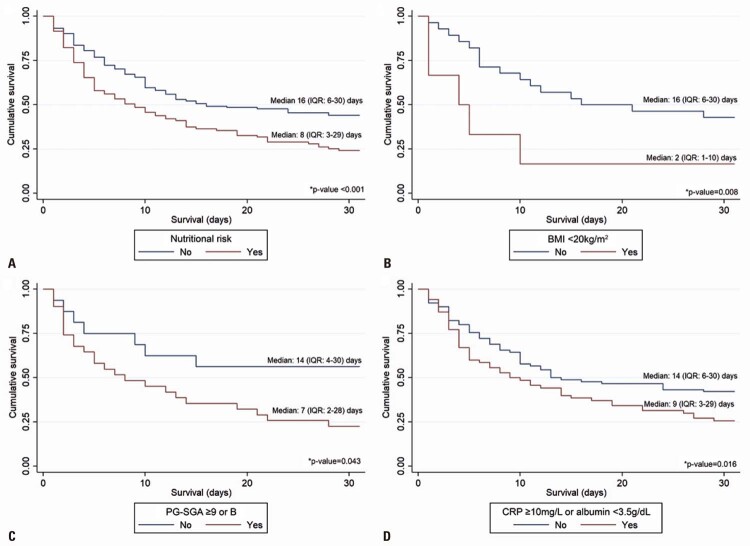
Survival curves of patients with cancer and COVID-19 diagnosis according to nutritional risk (A) and their indicators: BMI (B), PG-SGA score (C) and CRP or albumin level (D)

Survival was significantly lower in patients at nutritional risk (8 days; IQR: 3-29) than in patients not at nutritional risk (16 days; IQR: 6-30) (log rank p<0.001) ( Figure 3A ). Additionally, according to the Kaplan-Meier curves, survival was also lower when each nutritional risk indicator was evaluated separately: BMI <20kg/m ^2^ (2 *versus* 16 days; log-rank p=0.008; Figure 3B ); PG-SGA score ≥9 points or classification B (7 *versus* 14 days; log-rank p=0.043; Figure 3C ); albumin <3.5g/dL and/or CRP ≥10mg/L (9 *versus* 14 days; log-rank p=0.016; Figure 3D ).

* p value refers to a log-rank test.

IQR: interquartile range; BMI: body mass index; PG-SGA: Patient-generated Subjective Global Assessment; CRP: C-reactive protein.

According to the Cox regression analysis adjusted for multiple covariates, the presence of nutritional risk in patients with both cancer and COVID-19 was associated with a higher risk of 30-day mortality (HR: 1.42; 95%CI: 1.03-1.94).

In this multivariate model, only nutritional risk and poor performance status by KPS/ECOG-PS remained a risk factor for 30-day mortality (HR: 2.41; 95%CI: 1.71-3.42). In addition, nutritional risk was found to have good predictive power for prognostic discrimination (C-statistic: 0.744; 95%CI: 0.696-0.765) ( Table 2 ).

**Table 2:** Survival analysis and cox proportional hazard model of nutritional risk in the prediction of mortality

Variables	Survival (days)		Univariate		Multivariate		C-statistic

	Median (IQR)	p value*	HR (95%CI)	p value ^†^	HR (95%CI)	p value ^‡^	
Nutritional risk							
No	16 (6-30)	<0.001	Ref.	0.001	Ref.	0.030	
Yes	8 (3-29)		1.70 (1.25-2.32)		1.42 (1.03-1.94)		0.744
Adjusting factors							
Age (years)							
<60	13 (4-30)	0.378	Ref.	0.392			
> 60	10 (3-30)		1.15 (0.83-1.58)		-	-	-
Gender							
Male	14 (5-30)	0.669	Ref.	0.678			
Female	11 (4-30)		1.07 (0.78-1.47)		-	-	-
Tumor site							
Breast	5 (3-30)	0.082	1.76 (1.13-2.75)	0.013	-	-	-
Gastrointestinal tract	8 (3-30)		1.70 (1.08-2.66)	0.021	-	-	-
Gynecological	19 (4-30)		1.10 (0.65-1.86)	0.717	-	-	-
Head and neck	13 (4-30)		1.22 (0.60-2.52)	0.580	-	-	-
Lung	9 (3-30)		1.71 (0.96-3.05)	0.068	-	-	-
Bone and connective tissue	14 (6-30)		1.01 (0.40-2.56)	0.975	-	-	-
Others	22 (7-30)		Ref.		-	-	-
Cancer stage							
I and II	26 (7-30)	0.060	Ref.	0.061			
III and IV	10 (3-30)		1.51 (0.98-2.33)		-	-	-
Number of metastasis							
< 1	14 (5-31)	0.006	Ref.	0.008			
> 2	6 (3-31)		1.54 (1.12-2.11)				
Lung metastasis							
No	13 (4-30)	0.015	Ref.	0.019			
Yes	5 (3-21)		1.55 (1.07-2.34)		-	-	-
Surgery within 60 days							
No	10 (4-30)	0.074	Ref.	0.119			
Yes	30 (14-30)		0.41 (0.21-1.77)		-	-	-
Chemotherapy within 60 days							
No	10 (3-30)	0.109	Ref.	0.120			
Yes	16 (5-30)		0.77 (0.56-1.07)		-	-	-
Radiotherapy within 60 days							
No	12 (4-30)	0.606	Ref.	0.617			
Yes	9 (3-30)		1.14 (0.68-1.91)		-	-	-
*Diabetes mellitus*							
No	14 (5-30)	0.266	Ref.	0.281			
Yes	11 (4-30)		0.81 (0.54-1.19)		-	-	-
Hypertension							
No	10 (3-30)	0.500	Ref.	0.513			
Yes	13 (5-30)		0.90 (0.66-1.23)		-	-	-
Cardiovascular disease							
No	12 (4-30)	0.729	Ref.	0.737			
Yes	8 (3-30)		1.10 (0.63-1.90)		-	-	-
COPD							
No	11 (4-30)	0.501	Ref.	0.515			
Yes	10 (1-30)		1.27 (0.62-2.58)		-	-	-
ECOG-PS 3-4 or KPS≤40%							
No	30 (7-30)	<0.001	Ref.	<0.001	Ref.	<0.001	
Yes	8 (3-19)		2.62 (1.87-3.68)		2.41 (1.71-3.42)		-

* p value refers to a log-rank test; ^†^ p value refers to a cox proportional hazard model; ^‡^ p value refers to a multivariate cox proportional hazard model.

HR: hazard ratio; IQR: interquartile range; 95%CI: 95% confidence interval; COPD: chronic obstructive pulmonary disease; ECOG-PS: Eastern Cooperative Oncology Group performance status; KPS: Karnofsky Performance Status.

## DISCUSSION

The literature on the relationship between cancer-associated nutritional risk and COVID-19 outcomes is scant. In this study, patients at nutritional risk had significantly (almost 50%) higher of 30-day mortality than those not at nutritional risk. These findings suggest that patients with cancer at nutritional risk tend to have worse survival outcomes when infected with SARS-CoV-2.

In our results, prior nutritional risk in patients with cancer and COVID-19 varied according to: primary tumor site (higher prevalence in patients with bone and connective tissue (66.7%), gynecological (58.3%), and gastrointestinal tract (57.4%) cancer, all with p<0.050); disease stage (p=0.046); and performance status (p<0.001).

It is widely recognized that nutritional risk is common in patients with cancer, sometimes predating diagnosis, and previous evidence demonstrates that the prevalence of nutritional risk varies according to several factors. ^( 21 - 24 )^ In a multicenter study of 1,952 patients making their first appointment with an oncologist, 42.4% were found to be at nutritional risk according to their Mini Nutritional Assessment score. ^( 21 )^ Oliveira et al. ^( 33 )^ found 85.4% prevalence of nutritional risk among 1,039 patients with advanced cancer in palliative care, using the PG-SGA short form. Other studies have found varying prevalence of nutritional risk among non-cancer patients hospitalized with COVID-19 using different screening methods. ^( 13 - 16 )^

Cancer has been found to be associated with a higher risk of death in patients with COVID-19. ^( 5 - 8 )^ A study conducted by Fernandes et al. ^( 34 )^ among 411 patients with cancer and COVID-19 showed that 12.4% died and patients receiving palliative care were more likely to die (HR: 17.66; 95%CI: 3.13-99.59). In evaluating a subset of 109 patients with cancer from a cohort of 3,060 patients with COVID-19, Liang et al. ^( 20 )^ observed that 23 (21.1%) of them died, with a median time from admission to death of 7.62 days (IQR: 4.44-17.25). In addition, patients with cancer were at a higher risk of mortality than patients without cancer. Melo et al., ^( 35 )^ found 37.7% mortality in patients with cancer and COVID-19, and a significantly higher mortality risk in patients with higher serum CRP values (p=0.002). Pérez Camargo et al. ^( 36 )^ evaluated 121 cancer patients diagnosed with COVID-19, finding in the univariate analysis that hypoalbuminemia and nutritional impact symptoms were associated with lower survival. This is consistent with our finding that nutritional risk was associated with an increased risk of mortality. Our multivariate Cox analysis revealed that nutritional risk was an independent prognostic factor for 30-day mortality in cancer patients hospitalized for COVID-19 (HR: 1.42; 95%CI: 1.03-1.94), regardless of age, sex, tumor site, disease stage, comorbidities, or other recognized risk factors.

Additionally, our study found that nutritional risk had good discriminatory accuracy for predicting death (C-statistic: 0.744), albeit lower than that of the performance status scales (C-statistic: 0.745, data not shown). This was expected, considering that KPS and ECOG-PS are recognized as important scales for decision-making in cancer care and have good predictive accuracy for survival. ^( 37 - 40 )^ Therefore, even though nutritional risk preceded COVID-19 diagnosis, its predictive accuracy was similar to that of the performance status scales and other prognostic tools, such as the Palliative Prognostic Score (C-statistic: >0.79), the Palliative Prognostic Index (C-statistic: >0.75), ^( 41 )^ the Alternative International Prognostic Score-E (C-statistic: 0.70), ^( 42 )^ and the American Joint Committee on Cancer TNM Classification of Malignant Tumors (C-statistic: 0.74). ^( 43 )^

Published evidence about nutritional risk in patients with cancer and COVID-19 remains scant; however, a strong association has been found between lower survival and nutritional risk among non-cancer patients with COVID-19. ^( 13 , 18 )^ Nutritional risk and malnutrition are common in cancer, and can make patients more susceptible to severe respiratory tract infections. ^( 44 , 45 )^ Changes in nutritional status in patients with cancer differ from those found in patients with diseases of non-oncological etiology, and these changes are multifactorial. They result from the pathophysiological alterations caused by tumor-host interactions, such as increased pro-inflammatory activity, alteration of neuroendocrine signaling, protein catabolism, chemosensory alterations, decreased food intake, and greater occurrence of symptoms of nutritional impact. ^( 4 , 46 )^ The acute inflammatory process caused by infection concomitant with nutritional impairment in the host causes an increase in the pathogenicity of the infectious agent, resulting in worse clinical outcomes. ^( 44 , 45 )^ This could be explained by the fact that patients with cancer are in a state of metabolic stress characterized by adverse outcomes and increased complications. ^( 44 , 45 )^ Additionally, nutritional impact symptoms found in cancer, such as anorexia, anosmia, and weight loss, are also common with SARS-CoV-2 infection, potentially exacerbating the nutritional *deficits* already observed in patients with active malignancy. ^( 47 - 49 )^

The parameters used in our study to assess nutritional risk are recognized for this purpose, and their advantages and disadvantages should be considered, ^( 4 , 21 - 25 , 50 )^ as should the different features of malnutrition. As these were recorded prior to COVID-19 diagnosis, they may have changed before hospitalization in parallel with the disease progression. In addition, during the pandemic, nutritional status could have been impaired by difficulty in accessing supportive care and acquiring food due to the restrictions imposed to curb the spread of the virus. ^( 51 )^

Considering its convenience, low cost, and good ability to predict outcomes in patients with cancer, nutritional screening should be included as an integral part of the care approach for these patients, especially in the context of the COVID-19 pandemic. Because nutritional risk is a modifiable factor that can be reduced or controlled with early, individualized nutritional therapy after identifying nutritional risk could help prevent the disease from progressing and improve the prognosis for cancer patients infected with COVID-19. ^( 12 , 51 )^ This supports the idea that any nutritional derangements should be promptly and systematically managed in cancer patients with COVID-19, ^( 52 , 53 )^ and that nutritional care should be an integral part of care for these patients. However, future intervention trials are required to improve the current evidence.

Our findings cannot be considered conclusive due to an inherent bias caused by the study’s hospital-based retrospective nature and the fact that it did not include a control group of non-cancer patients with malnutrition and COVID-19, since it was carried out in a specialized cancer hospital. However, the data still highlight the importance of frequent nutritional screening to enable malnutrition in cancer patients to be detected and treated early in order to improve COVID-19 outcomes.

Another limitation of our study is that high CRP concentration is considered both a nutritional prognostic marker ^( 4 )^ and an associated factor of mortality for cancer and COVID-19 patients. ^( 35 , 38 )^ Although the CRP values considered in this study were evaluated before COVID-19 diagnosis, this marker is used to assess the magnitude (acute or chronic) of systemic inflammatory response, ^( 5 )^ so its use could be considered a bias. Finally, our outcome assessment may be flawed because some of the discharged patients may have been readmitted elsewhere with severe illness and died after the follow-up period.

These results reinforce several important considerations for clinical care and emphasize the importance of nutritional care in patients with cancer. The prevention, screening, and treatment of nutritional risk should be included in the routine care of cancer patients with COVID-19.

## CONCLUSION

This study demonstrates that the presence of prior nutritional risk is related to poor prognosis in patients with cancer and COVID-19. Since nutritional risk is a potentially modifiable factor, nutrition could be an important element for improving the clinical outcomes of these patients in the context of the pandemic.
